# Efficacy and safety of short-acting β-blockers in patients with sepsis-associated cardiac dysfunction: a systematic review and meta-analysis of randomized controlled trials

**DOI:** 10.3389/fcvm.2025.1665466

**Published:** 2025-09-09

**Authors:** Min'an Zheng, Jin Wang, Pingchang Xie, Shijun Guo, Benjian Chen, Zhuogen He, Guoyan Yao

**Affiliations:** ^1^ Department of Emergency, The Second Affiliated Hospital of Guangzhou University of Chinese Medicine, Guangzhou, Guangdong, China; ^2^ Department of Medical, The Second Affiliated Hospital of Guangzhou University of Chinese Medicine, Guangzhou, Guangdong, China; ^3^ Department of Radiology, The Second Affiliated Hospital of Guangzhou University of Chinese Medicine, Guangzhou, Guangdong, China

**Keywords:** ultra-rapid β-blockers, sepsis, cardiac dysfunction, healing efficacy, safety

## Abstract

**Background:**

The role of ultra-rapid β-blockers in sepsis-associated cardiac dysfunction remains controversial, with conflicting evidence regarding mortality benefits and safety concerns in hemodynamically unstable patients.

**Methods:**

This study retrieved relevant reports on randomized controlled trials of ultra-rapid β-blockers conducted for adult patients with sepsis-associated cardiac dysfunction, up to and including the date of May 30, 2025, from the databases of PubMed, Web of Science, Cochrane Library and Embase. Primary outcomes were 28-day mortality and adverse events; secondary outcomes included heart rate control and mean arterial pressure (MAP) at 48 h. Random-effects models calculated risk ratios (RR) or standardized mean differences (SMD) with 95% confidence intervals (CI). Heterogeneity was assessed using I² statistics.

**Results:**

Eight studies reported 28-day mortality, showing no significant reduction with ultra-rapid β-blockers (RR, 0.84, 95% CI: 0.67–1.06; *P* = 0.15; I² = 54%). Safety data from four studies indicated no increased adverse events (RR, 1.04, 95% CI: 0.82–1.33; *P* = 0.72; I² = 0%). Paradoxically, ultra-rapid β-blockers were associated with worse heart rate control (RR, 1.51, 95% CI: 1.00–2.29; *P* = 0.05). MAP at 48 h showed no intergroup difference (SMD, −0.85, 95% CI: −2.24–0.54).

**Conclusion:**

ultra-rapid β-blockers demonstrate an acceptable safety profile without compromising hemodynamic stability but fail to reduce 28-day mortality in sepsis-associated cardiac dysfunction patients. The inferior heart rate control suggests potential physiological incompatibility in this population. Precision targeting based on adrenergic activity and cardiac phenotyping warrants investigation.

## Introduction

Sepsis, one of the leading causes of death in critically ill patients worldwide, is characterized by life-threatening organ dysfunction triggered by an uncontrolled host response to infection (1, 2). When sepsis involves the circulatory system, it can induce or exacerbate cardiac dysfunction (i.e., sepsis-associated cardiac dysfunction, refers to newly emerging reversible heart failure during the course of sepsis, characterized by a decrease in left ventricular ejection fraction (LVEF < 50%) or elevated cardiac injury markers (cTnI > 0.4 ng/ml), and must exclude patients with chronic heart failure), creating a critical state with complex pathophysiological mechanisms and a very poor clinical prognosis (3, 4). These patients not only face a high risk of death due to sepsis itself (in-hospital mortality can be as high as 40%–60%) but are also caught in a vicious circle of hemodynamic collapse due to the rapid deterioration of cardiac function. The severity of sepsis is twofold: on the one hand, sepsis releases a storm of inflammatory mediators and cytokines that can directly inhibit myocardial contractility and impair ventricular function, leading to “septic cardiomyopathy” (5); on the other hand, although the compensatory activation of the sympathetic nervous system (SNS) can temporarily maintain perfusion, the continuous excessive catecholamine release can cause tachycardia, a dramatic increase in myocardial oxygen consumption, calcium regulation disorders, and direct cardiotoxicity, which can accelerate cardiac failure and significantly increase the risk of multi-organ failure and death (6, 7).

The treatment of sepsis-associated cardiac dysfunction is highly urgent and complex. Conventional supportive therapies (e.g., fluid resuscitation, vasoactive drugs) can partially correct the hemodynamic disturbances but are often ineffective in controlling the vicious cycle of sympathetic overactivation (8, 9). β-blockers, as a class of negative inotropic and negative frequency drugs, are necessary because of their potential dual pathological interventions: by antagonizing β1 receptors, reducing persistently elevated heart rate, decreasing myocardial oxygen consumption, and shorten the ventricular filling time; in addition, they may also alleviate adrenergic-mediated myocardial cell damage, calcium overload, and metabolic disorders, thereby protecting myocardial function and potentially improving long-term prognosis (10, 11); However, when patients are in a state of metabolic hyperactivity and high stress, the use of ultra-rapid β-blockers may cause hypotension or mask signs of hypoperfusion by inhibiting compensatory cardiac output. These potential risks raise questions about the safety of their clinical application (12).

In view of this, this systematic review and Meta-analysis aims to comprehensively integrate the existing clinical research evidence and quantitatively assess the efficacy and safety of ultra-rapid β-blockers in the treatment of sepsis-associated cardiac dysfunction patients. The results of the study will provide a key evidence-based basis for clinical development of individualized treatment strategies and design of high-quality prospective trials.

## Methods

### Search strategy

The search was performed using the terms “sepsis” or “heart failure” or “cardiac insufficiency” or “β-blocker” up to 30 May 2025 in the Cochrane Library, Web of science and Embase databases. Duplicate items were excluded from the search results. The reference lists of articles were also reviewed for this study. Two researchers independently reviewed the titles, keywords, abstracts, and full text of all identified articles to retain those that met the screening criteria. Any doubts about the inclusion of articles were resolved by a third researcher after discussion and consensus. The detailed database search strategy is provided in Supplementary Table S1.

### Criteria for study selection

The inclusion criteria were defined as follows: i. Study type: randomized controlled trial (RCT). ii. Study population: patients diagnosed with sepsis and presenting with at least one of the following conditions: definite reduction in left ventricular ejection fractions (LVEF); significantly elevated cardiac biomarkers (BNP/NT-proBNP, Troponin); persistent, uncontrollable sinus tachycardia (heart rate >110–120 bpm unresponsive to volume resuscitation and basal therapy) or tachyarrhythmias that require treatment (13). Although the search terms include traditional terms such as “heart failure,” the studies ultimately included in the analysis all meet the modern definition of sepsis-associated heart failure (sepsis-induced + acute onset + abnormal objective indicators), which is fundamentally different from traditional chronic heart failure. iii. Intervention: β-blocker therapeutic intervention. iv. Outcome Indicators: a. Primary Outcome: 28-day mortality, incidence of adverse events. b. Secondary outcomes: heart rate control effect, mean arterial pressure (MAP). v. Language of articles: Only English and Chinese articles were included.

Exclusion criteria were as follows: i. Duplicate literature studies, systematic evaluations, reviews and case reports. ii. Animal studies, studies in children or adolescents (<18 years old). iii. Non-β-blockers. iv. Studies with incomplete data or unclear methodology were excluded.

### Data extraction

Two researchers independently reviewed titles and abstracts to identify studies relevant to the article topic. Two independent reviewers fully downloaded and assessed the eligible literature. Two independent reviewers extracted data from the included studies. Extracted data included 1. authors; 2. year of publication; 3. participants (age, gender, sample size); 4. intervention characteristics; and 6. study outcomes. Any discrepancies that arose during the process were resolved by consensus among the assessors, with a third assessor consulted if necessary.

### Risk of bias assessment

The reviewers used the revised Cochrane Risk of Bias tool for randomized trials to assess the risk of bias (RoB 2.0) (14). This study evaluated the risk of bias based on aspects such as bias during the randomization process, deviation from the expected intervention measures, the situation of missing result data, the measurement of results, and the selection of intervention subjects. It classified the risk of bias into four levels: low, possibly low, possibly high, or high. The disagreement was resolved through discussion, and in case of necessity, a third party would make the final decision.

### Statistical analysis

The Mantel-Haenszel random effects model was used for the statistical analysis of binary classification results, and the inverse variance random effects model was used for the statistical analysis of continuous results. The risk ratio (RR) or standardized mean difference (SMD) was presented in the form of point estimates, along with a 95% confidence interval and *P* value. For data presented in the form of median and interquartile range, the median and interquartile range were converted to mean and standard deviation to obtain the combined RR and SMD. The Mantel-Haenszel *x^2^* test and I^2^ statistic (the proportion of total variation explained by heterogeneity) were used to investigate statistical heterogeneity (15). All analyses were completed using RevMan 5.1.6.

## Results

### Study selection and study characteristics

**Figure 1:**
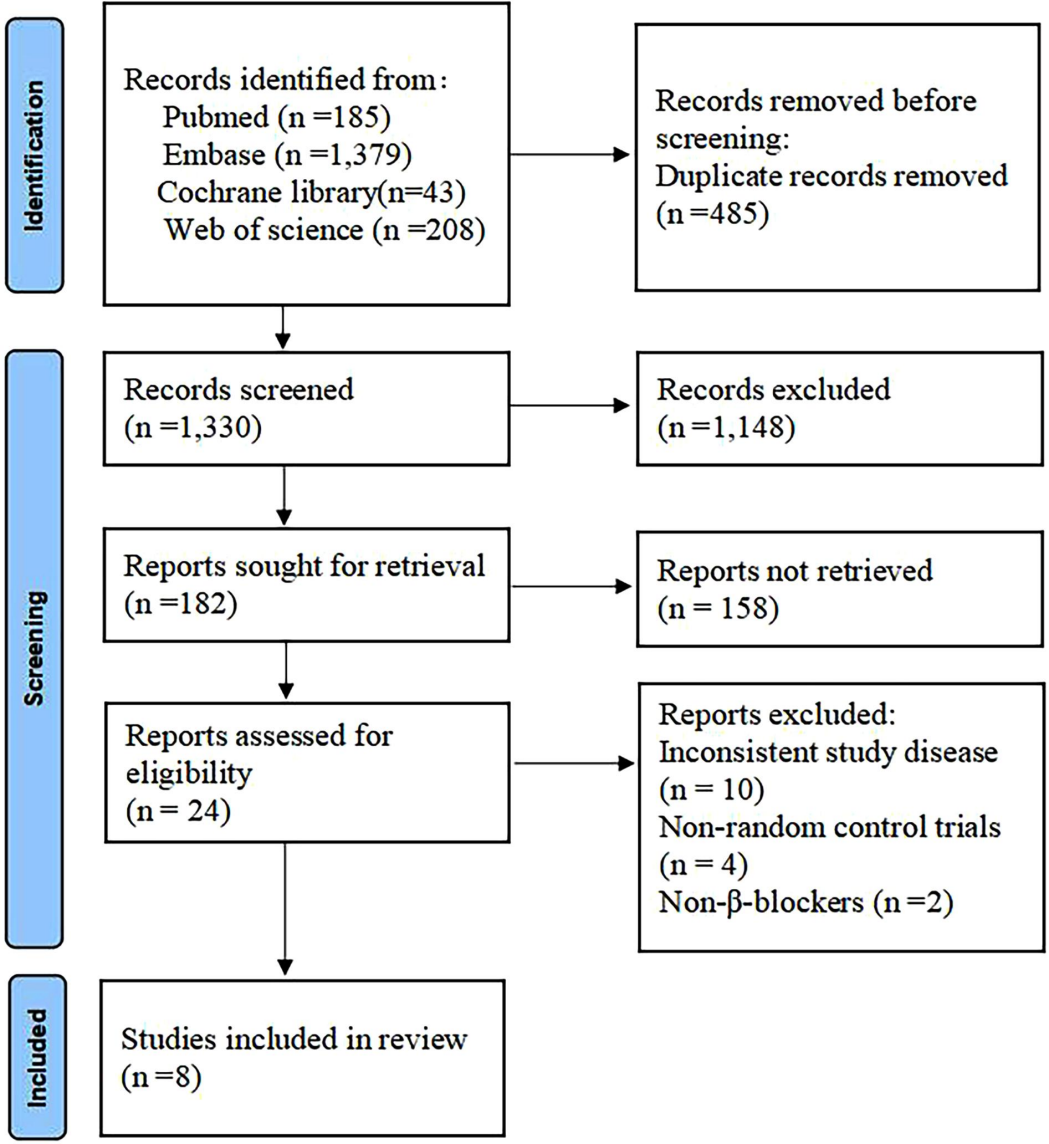
Study screening flowchart.

A total of 1,815 articles were retrieved through the database search (Figure 1). Of these, 485 duplicates were removed, and 1,148 records were excluded due to title and abstract incompatibility. Ultimately, a total of eight studies were included in this meta-analysis, with a combined sample size of 871 patients (16–23). Table 1 presents the basic characteristics of the included studies. The sample size ranged from 24–196. All eight trials reported 28-day mortality, six reported the effect of heart rate control, five investigated hemodynamic parameters, and only four reported the incidence of adverse events. Four trials were single-center RCTs in China, two were single-center and multicenter RCTs in the United Kingdom, one trial was a multicenter RCT in Japan, and one trial was conducted in multicenter RCT conducted in Europe. The mean/median age of the participants was reported to be between 34 and 68 years, with 57%–67% of them being male.

**Table 1:** Characteristics of studies included in the meta-analysis.

Study	Country	Setting	Sample Size	Age, y	Men, %	Cardiac abnormalities	Intervention	Timing of β-blocker intervention	Initial Dose	Titration Protocol	Discontinuation Criteria	Primary Outcome	Secondary Outcome
Kakihana 2020 (16)	Japan	Multicenter	151	67.1	60	Atrial fibrillation or sinus tachycardia (>100 bpm)	Landiolol	Early stage of resuscitation	1.0 μg/kg/min	increased by 1 μg/kg/min q15–20 min until Heart rate < 95 bpm	SBP ≥ 20% from baseline or Heart rate <60 bpm	28-day mortality	Heart rate control effects
												Adverse event rates	
Liu 2019 (17)	China	Single center	100	57	57	Tachycardia (>100 bpm)	Esmolol	Early stage of resuscitation	5.0 mg/h	increased by 50 mg/h every 20min	Continue administration of esmolol for 7 days	28-day mortality	NR
Lu 2025 (18)	China	Single center	24	59	67	Sinus tachycardia (>95 bpm)	Esmolol	Early stage of resuscitation	50 ml of solution (10 mg/ml)	Load dose 20 mg (1 min) + maintenance dose 25 mg/h	Suspend infusion when Heart rate < 80 bpm	28-day mortality	Heart rate control effects
													48 h MAP
Morelli 2013 (19)	England	Single center	154	68	69.4	Tachycardia (>100 bpm)	Esmolol	Early stage of resuscitation	25 mg/ml	50 mg/h (20 min)	Maintain the heart rate threshold (<80 bpm)	28-day mortality	Heart rate control effects
													48 h MAP
Rehberg 2024 (20)	European	Multicenter	196	64.4	60.2	Persistent tachycardia (>100 bpm)	Landiolol	Early stage of resuscitation	1.0 μg/kg/min	Increase by 1 μg/kg/min every 20 min (up to a maximum of 40 μg/kg/min) until the heart rate stabilizes at 80–94 bpm for 15 min	Maintain the heart rate threshold (<90 bpm)	28-day mortality	Heart rate control effects
												Adverse event rates	
Tony 2023 (21)	England	Multicenter	126	55.6	58.7	Tachycardia (heart rate ≥ 95 beats per minute)	Landiolol	Early stage of resuscitation	1.0 μg/kg/min.	Increase by 1 μg/kg/min every 15 min until the heart rate stabilizes at 80–94 bpm	Maintain the heart rate threshold (<80 bpm)	28-day mortality	Heart rate control effects
												Adverse event rates	
													48 h MAP
Wang 2015 (23)	China	Single center	60	34	65	Tachycardia (>100 bpm)	Esmolol	NR	NR	NR	NR	28-day mortality	Heart rate control effects
												Adverse event rates	
													48 h MAP
Wang 2017 (22)	China	Single center	60	64.9	65	Tachycardia (>100 bpm)	Esmolol	Early stage of resuscitation	0.05 mg/kg/h.	Increase by 0.001 mg/kg/h every 5 min (up to a maximum of 0.2 mg/kg/h) until the heart rate stabilizes at 95 bpms for 5 min	Maintain the heart rate threshold (<95 bpm)	28-day mortality	48 h MAP

NR, not reported; MAP, mean arterial pressure; SBP, systolic blood pressure.

### Risk of bias

**Figure 2:**
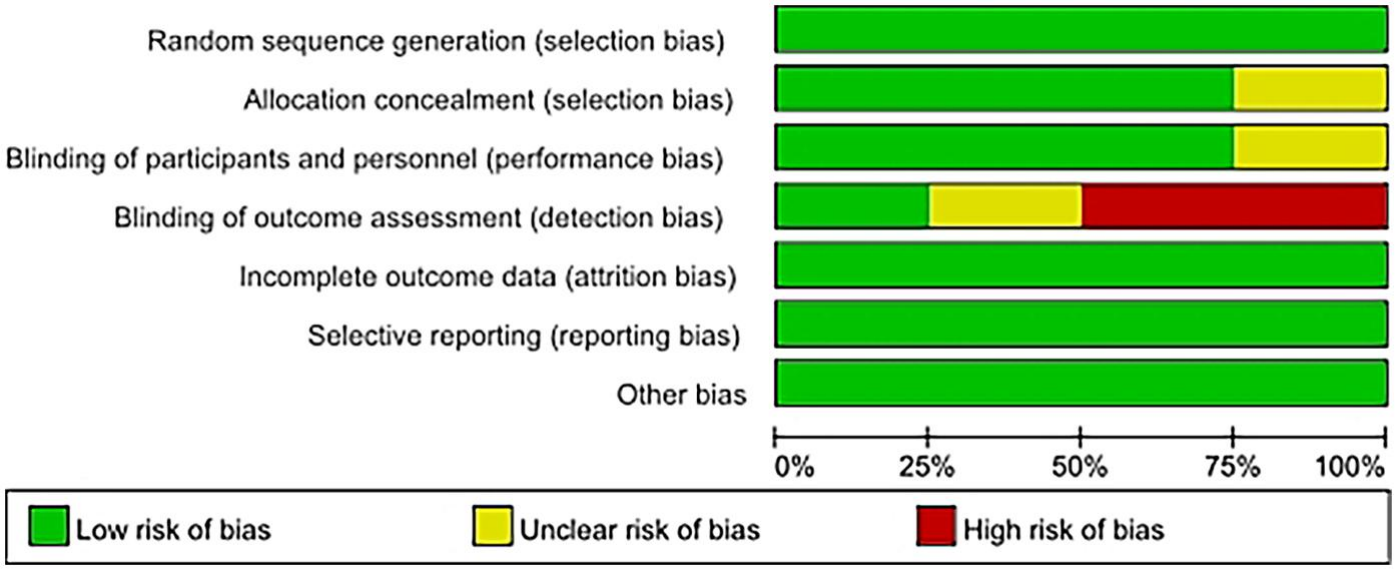
Risk of bias graph.

**Figure 3:**
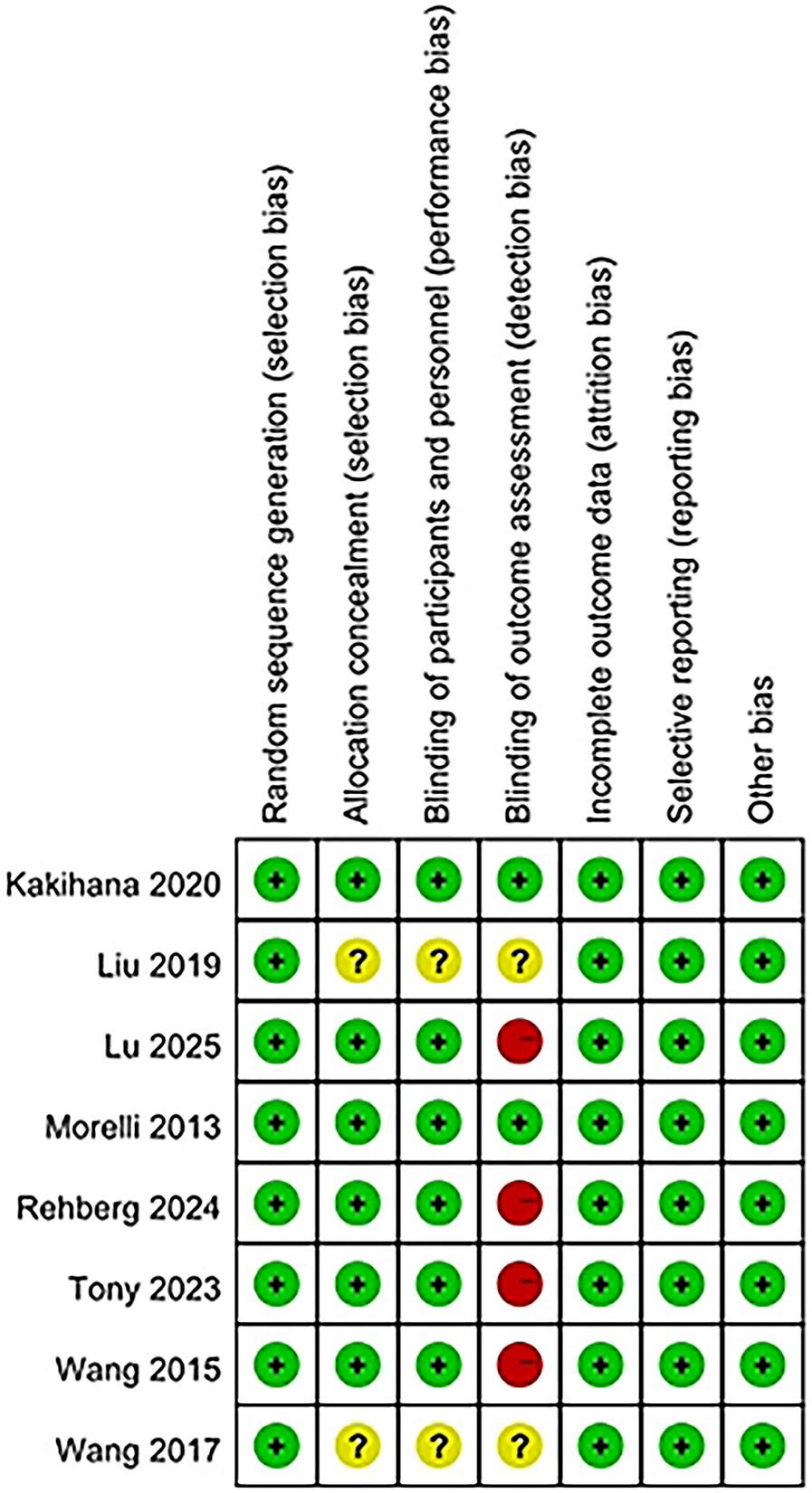
Risk of bias summary.

The risk of bias for eight studies was evaluated, and detailed information can be found in the bias risk summary table (Figure 2) and the bias risk chart (Figure 3). Six studies had a low risk of bias in terms of allocation concealment (selection bias); while two other studies could not be evaluated due to the lack of relevant information. Six studies were at low risk of blinding of participants and staff (performance bias). Two other studies could not be evaluated regarding the blinding of participants and personnel. Four studies had a high risk of bias in terms of outcome assessment blinding, while two studies had a low risk of bias in this regard, and two other studies could not be evaluated regarding the outcome assessment blinding situation due to the lack of relevant information. Regarding other biases, the risk was low for all eight studies.

## Primary outcomes

### 28-day mortality rate

**Figure 4:**
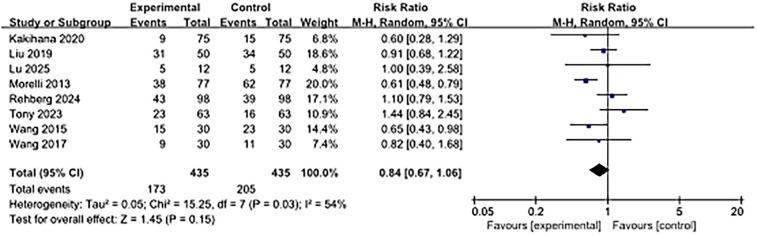
Forest plot of 28-day mortality.

As shown in Figure 4, 28-day mortality was reported in eight studies. The use of ultra-rapid β-blockers in patients with sepsis-associated cardiac dysfunction did not show a significant association with lower 28-day mortality (RR, 0.84; 95% CI: 0.67–1.06; *P* = 0.15). Moderate heterogeneity was observed (I^2^ = 54%).

### The incidence of adverse events in patients

**Figure 5:**
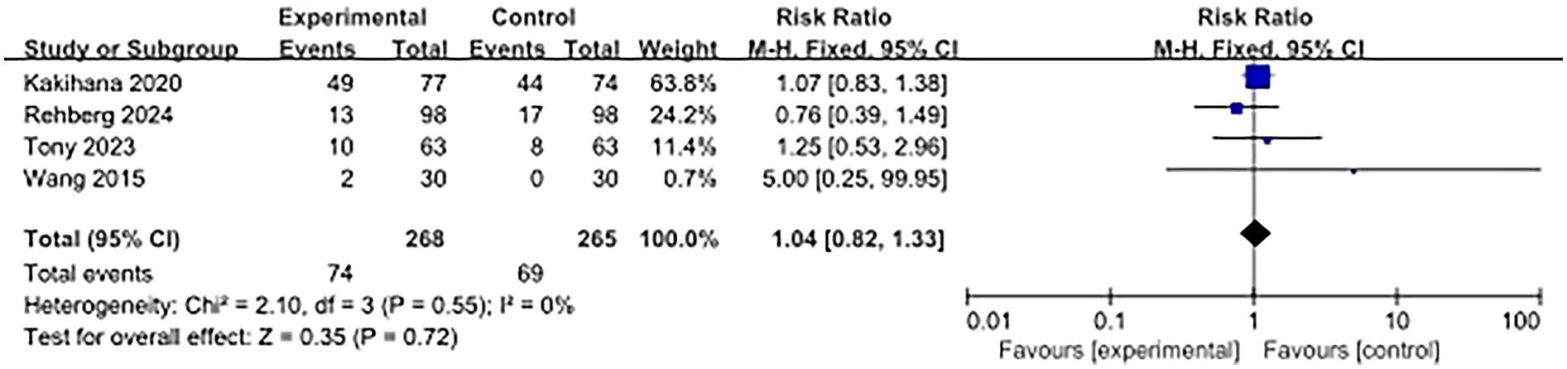
Forest plot of adverse event rates.

Four studies reported adverse events. The use of ultra-rapid β-blockers in patients with sepsis-associated cardiac dysfunction did not show a significant association with the occurrence of adverse events (RR, 1.04; 95% CI: 0.82–1.33; *P* = 0.72). No heterogeneity was observed (I^2^ = 0%) (Figure 5).

## Secondary outcomes

### Heart rate control effect

**Figure 6:**
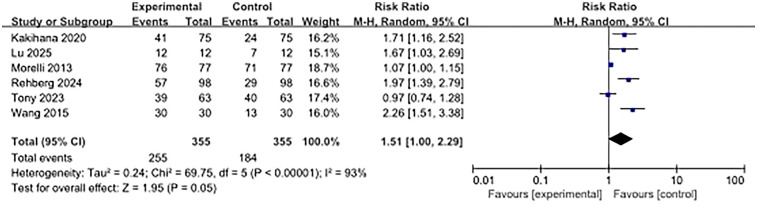
Forest plot of heart rate control effects.

Six articles examined the heart rate control effect. The results showed that the heart rate control effect of the beta-blocker group was significantly worse than that of the control group (RR, 1.51; 95% CI, 1.00–2.29; *P* = 0.05). High heterogeneity was observed (I^2^ = 93%) (Figure 6).

### Mean arterial pressure

**Figure 7:**
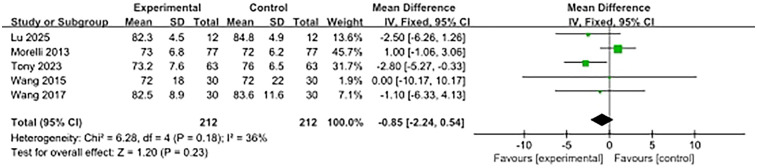
Forest plot for 48 h MAP.

Five studies reported on MAP at 48 h after enrolment, and there was no significant difference between the β-blocker group and the control group (SMD, −0.85; 95% CI, −2.24–0.54). Moderate heterogeneity was observed (I^2^ = 36%) (Figure 7).

### Publication bias

In this study, the publication bias of the primary outcome measure (28-day mortality rate) was evaluated by visually inspecting the symmetry of the funnel plot. The results indicated that the funnel plot was symmetrical, and there was no possibility of publication bias (Supplementary Figure S1).

## Discussion

This meta-analysis provides a comprehensive evaluation of ultrashort-acting β-blockers (esmolol/landiolol) in sepsis-associated cardiac dysfunction, revealing critical insights into their hemodynamic effects and clinical outcomes. The findings challenge conventional assumptions while highlighting the complexity of adrenergic modulation in critical illness.

The reduction in the 28-day mortality rate did not reach statistical significance. Although the point estimate suggested a 16% relative risk reduction, the confidence interval included the null value (RR = 1), indicating that the result may have been due to random variation. However, from a pathophysiological perspective, beta-blockers have a dual mechanism of action in sepsis: they provide potential cardiac protection by reducing myocardial oxygen demand and alleviating catecholamine toxicity, while also impairing compensatory mechanisms in distributive shock (24, 25). The observed moderate heterogeneity (I² = 54%) may be due to differences in patients' hemodynamic phenotypes (e.g., cardiogenic shock vs. vasodilatory shock). Although the current data exclude a clinically significant effect of a mortality reduction >33%, smaller effects may still exist and require validation with a larger sample size.

The adverse event profile with null heterogeneity is arguably the most significant finding. It confirms the hemodynamic safety margin of ultrashort-acting agents in this high-risk population (26). Their rapid offset enables precise titration, mitigating traditional concerns about ultra-rapid β-blockers in shock states (27). This pharmacodynamic advantage likely underpins the preserved MAP at 48 h, suggesting these agents can be administered without compromising perfusion pressure when hemodynamic monitoring is available. Contrary to mechanistic expectations, β-blocker use associated with inferior heart rate control. This counterintuitive result exposes fundamental knowledge gaps: In sepsis-associated cardiac dysfunction, tachycardia may be essential to maintain cardiac output in the context of reduced stroke volume (28, 29). Blunting this reflex without concomitant inotropic support could precipitate decompensation. Rapid drug discontinuation in studies with protocolized limited-duration infusions may trigger paradoxical tachycardia. Current titration protocols (often targeting HR < 100 bpm) might inadequately address the hyperadrenergic state of sepsis, leading to underdosing and apparent “failure” of rate control.

This study shares continuity with the meta-analyses conducted by Hasegawa D et al. and Perala A et al. in terms of the core research question, both focusing on the application value of ultra-rapid β-blockers in patients with sepsis and showing a consistent trend of benefit in terms of 28-day mortality outcomes (30, 31). However, previous studies only used 28-day mortality as a single outcome. This study expanded the evaluation framework to a three-dimensional system of “survival-function-safety,” adding heart rate control efficacy, hemodynamic stability, and adverse event incidence, addressing the clinical need for multi-dimensional efficacy and safety assessment; Second, this study exclusively included randomized controlled trials, reducing confounding bias by excluding observational studies, thereby providing high-level evidence support. In contrast, previous studies did not strictly restrict study types, leading to variations in evidence strength. This design optimization not only validated the core conclusions but also enhanced the clinical relevance of the findings through rigorous study type screening and expanded outcome dimensions.

Although the use of ultra-rapid β-blockers in sepsis has been explored in multiple randomized controlled trials, existing studies have the following limitations: First, most trials only report a single outcome (e.g., 28-day mortality) and lack systematic analysis of functional indicators such as heart rate control and hemodynamics; Second, different trials have inconsistent definitions of the “sepsis-associated cardiac dysfunction” subgroup, leading to fragmented evidence. This study systematically integrated eight high-quality randomized controlled trials to establish a three-dimensional outcome framework of “survival-function-safety” for the first time, consolidating dispersed single-trial data into a multidimensional evidence chain.

Interpretation of this study requires careful consideration of the following limitations: first, the limited number of original studies included (only 8 assessing mortality and 4 reporting adverse events) may reduce statistical power and increase the risk of type II error, especially for key outcomes such as 28-day mortality; second, significant clinical heterogeneity (I² = 54%) stemmed from differences in baseline patient characteristics (e.g., sepsis etiology, cardiac dysfunction severity, concomitant therapy) and inconsistent intervention regimens (β-blocker dose, titration rate, target heart rate), which were not adequately corrected for; finally, the lack of individual patient data limited the ability of subgroup analyses (e.g., distinguishing between preserved vs. reduced cardiac dysfunction with preserved ejection fraction) to identify populations of potential benefit. Furthermore, this analysis only confirms that ultra-rapid beta-blockers do not lower mean arterial pressure, but this does not equate to overall hemodynamic stability. Future studies need to systematically monitor changes in vasoactive drug requirements (such as norepinephrine equivalent doses), cardiac function parameters (cardiac index, stroke volume), and tissue perfusion indicators (lactate, ScvO₂) to comprehensively assess safety.

Based on current evidence, ultra-rapid β-blockers for patients with sepsis-associated cardiac dysfunction, although demonstrating an acceptable safety profile (no significant increase in risk of adverse events, blood pressure stabilization at 48 h), failed to significantly reduce 28-day mortality and were associated with worse heart rate control. This paradox suggests that using heart rate alone as a therapeutic target may be insufficient or even harmful, especially in pathological states where compensatory tachycardia maintains cardiac output. Future studies should focus on precise patient selection (e.g., high sympathetic tone subgroups), optimizing ICU hemodynamic management (integrating cardiac output monitoring), and exploring combination therapeutic strategies (e.g., coadministration of positive inotropic medications) to reevaluate the balance of risk-benefit of ultra-rapid β-blockers in this complex population.

## Data Availability

The original contributions presented in the study are included in the article/Supplementary Material, further inquiries can be directed to the corresponding author.
