# Supplementary material for: Efficacy and safety of short-acting β-blockers in patients with sepsis-associated cardiac dysfunction: a systematic review and meta-analysis of randomized controlled trials
Source: Front Cardiovasc Med. 2025 Sep 9;12:1665466. doi: 10.3389/fcvm.2025.1665466 (PMC12454437; doi:10.3389/fcvm.2025.1665466)
Supplement: Supplementary file 1 [file Datasheet1.pdf]

Table S1 Search strategy

PubMed

Set	Search terms	Search type	Results
#1	"Sepsis"[Mesh] OR "Bloodstream Infection*"[tiab] OR "Infection, Bloodstream"[tiab] OR "Septicemia*"[tiab] OR "Blood Poisoning*"[tiab] OR "Poisonings, Blood"[tiab] OR "Severe Sepsis"[tiab] OR "Pyemia*"[tiab] OR "Pyohemia"[tiab]	Advanced	170,743
#2	"Heart Failure"[Mesh] OR "Cardiac Failure"[tiab] OR "Heart Decompensation"[tiab] OR "Congestive Heart Failure"[tiab] OR "Heart Failure, Right-Sided"[tiab] OR "Right-Sided Heart Failure"[tiab] OR "Heart Failure, Left-Sided"[tiab] OR "Left-Sided Heart Failure"[tiab] OR "Myocardial Failure"[tiab] OR "Cardiac insufficiency"[tiab] OR "Myocardial dysfunction"[tiab] OR "HF"[tiab]	Advanced	237,866
#3	"Adrenergic beta-Antagonists"[Mesh] OR "beta-Antagonists, Adrenergic"[tiab] OR "Adrenergic beta-Antagonist"[tiab] OR "Adrenergic beta-Receptor Blockader"[tiab] OR "beta-Receptor Blockader, Adrenergic"[tiab] OR "beta-Adrenergic Antagonist"[tiab] OR "Antagonist, beta-Adrenergic"[tiab] OR "beta Adrenergic Antagonist"[tiab] OR "beta-Adrenoceptor	Advanced	47,198

	Antagonists"[tiab] OR "beta Adrenoceptor Antagonists"[tiab] OR "Adrenergic beta Receptor Blockaders"[tiab] OR "Blockaders, Adrenergic beta-Receptor"[tiab] OR "beta Adrenergic Receptor Blockaders"[tiab] OR "beta-Adrenergic Blocking Agents"[tiab] OR "beta Adrenergic Blockers"[tiab] OR "beta Adrenoceptor Antagonist"[tiab] OR "bBlocker, beta-Adrenergic"[tiab]		
#4	#1 AND #2 AND #3	Advanced	185

Embase

**Set**	**Search terms**	**Search type**	**Results**
#1	'sepsis'/exp OR 'abdominal sepsis':ti,ab,kw OR 'focal sepsis':ti,ab,kw OR 'intraabdominal sepsis':ti,ab,kw OR 'sepsis syndrome':ti,ab,kw OR 'septic disease':ti,ab,kw OR 'sepsis':ti,ab,kw	Advanced	441,160
#2	'heart failure'/exp OR 'backward failure, heart':ti,ab,kw OR 'cardiac backward failure':ti,ab,kw OR 'cardiac decompensation':ti,ab,kw OR 'cardiac failure':ti,ab,kw OR 'cardiac incompetence':ti,ab,kw OR 'cardiac insufficiency':ti,ab,kw OR 'cardiac stand still':ti,ab,kw OR 'cardial decompensation':ti,ab,kw OR 'cardial insufficiency':ti,ab,kw	Advanced	83,941

	OR 'chronic heart failure':ti,ab,kw OR 'chronic heart insufficiency':ti,ab,kw OR 'decompensatio cordis':ti,ab,kw OR 'decompensation, heart':ti,ab,kw OR 'heart backward failure':ti,ab,kw OR 'heart decompensation':ti,ab,kw OR 'heart incompetence':ti,ab,kw OR 'heart insufficiency':ti,ab,kw OR 'insufficientia cardis':ti,ab,kw OR 'myocardial failure':ti,ab,kw OR 'myocardial insufficiency':ti,ab,kw OR 'heart failure':ti,ab,kw		
#3	'beta adrenergic receptor blocking agent'/exp OR 'adrenergic beta antagonists':ti,ab,kw OR 'adrenergic beta-antagonists':ti,ab,kw OR 'antiadrenergics, beta blocking':ti,ab,kw OR 'beta adrenergic antagonist':ti,ab,kw OR 'beta adrenergic blocker':ti,ab,kw OR 'beta adrenergic blockers':ti,ab,kw OR 'beta adrenergic blocking agent':ti,ab,kw OR 'beta adrenergic blocking drug':ti,ab,kw OR 'beta adrenergic receptor antagonist':ti,ab,kw OR 'beta adrenergic receptor blocker':ti,ab,kw OR 'beta adrenoceptor antagonist':ti,ab,kw OR 'beta adrenoceptor blocker':ti,ab,kw OR 'beta adrenoceptor blocking agent':ti,ab,kw OR 'beta adrenoceptor blocking drug':ti,ab,kw OR 'beta adrenolytic':ti,ab,kw OR 'beta adrenolytic agent':ti,ab,kw OR 'beta antagonist':ti,ab,kw OR 'beta antiadrenergic agent':ti,ab,kw OR 'beta blocker':ti,ab,kw OR 'beta blocking adrenergic agent':ti,ab,kw OR 'beta blocking agent':ti,ab,kw OR 'beta blocking drug':ti,ab,kw	Advanced	377,855

	OR 'beta receptor adrenergic blocking agent':ti,ab,kw OR 'beta receptor blocker':ti,ab,kw OR 'beta receptor blocking agent':ti,ab,kw OR 'beta sympathicolytic agent':ti,ab,kw OR 'beta sympathicolytics':ti,ab,kw OR 'beta sympatholytic agent':ti,ab,kw OR 'betasympatholytic agent':ti,ab,kw OR 'beta adrenergic receptor blocking agent':ti,ab,kw OR 'Cardiac insufficiency':ti,ab,kw OR 'Myocardial dysfunction':ti,ab,kw		
#4	#1 AND #2 AND #3	Advanced	1,379

Web of Science

Set	Search terms	Search type	Results
#1	TS=("sepsis" OR "abdominal sepsis" OR "focal sepsis" OR "intraabdominal sepsis" OR "sepsis syndrome" OR "septic disease" OR "sepsis")	Advanced	248,138
#2	TS=("Heart Failure" OR "Cardiac Failure" OR "Heart Decompensation" OR "Congestive Heart Failure" OR "Heart Failure, Right-Sided" OR "Right-Sided Heart Failure" OR "Heart Failure, Left-Sided" OR "Left-Sided Heart Failure" OR "Myocardial Failure" OR "HF")	Advanced	636,065
#3	TS=("Adrenergic beta-Antagonists" OR "beta-Antagonists, Adrenergic" OR "Adrenergic beta-Antagonist" OR "Adrenergic	Advanced	62,036

	beta-Receptor Blockader" OR "beta-Receptor Blockader, Adrenergic" OR "beta-Adrenergic Antagonist" OR "Antagonist, beta-Adrenergic" OR "beta Adrenergic Antagonist" OR "beta-Adrenoceptor Antagonists" OR "beta Adrenoceptor Antagonists" OR "Adrenergic beta Receptor Blockaders" OR "Blockaders, Adrenergic beta-Receptor" OR "beta Adrenergic Receptor Blockaders" OR "beta-Adrenergic Blocking Agents" OR "beta Adrenergic Blockers" OR "beta Adrenoceptor Antagonist" OR "Blocker, beta-Adrenergic" OR "Cardiac insufficiency" OR "Myocardial dysfunction")		
#4	#1 AND #2 AND #3	Advanced	43

Cochrane Library

**Set**	**Search terms**	**Search type**	**Results**
#1	MeSH descriptor: [Sepsis] explode all trees	Advanced	6,552
#2	("Pyohemia" OR "Pyemia" OR "Pyaemia" OR "Severe Sepsis" OR " Infection, Bloodstream" OR "Poisoning, Blood" OR "Septicemias" OR "Severe Sepsis"):ti,ab,kw	Advanced	1,538
#3	#1 OR #2	Advanced	7,381
#4	MeSH descriptor: [Heart Failure] explode all trees	Advanced	14,864
#5	("Heart Failure, Congestive" OR "Congestive Heart Failure" OR	Advanced	7,683

	"Cardiac Failure" OR "Right-Sided Heart Failure" OR "Heart Failure, Right Sided" OR "Heart Failure, Right-Sided" OR "Right Sided Heart Failure" OR "Heart Failure, Left Sided" OR "Heart Failure, Left-Sided" OR "Left-Sided Heart Failure" OR "Myocardial Failure" OR "Heart Decompensation" OR "Cardiac insufficiency" OR "Myocardial dysfunction"):ti,ab,kw		
#6	#4 OR #5	Advanced	19,935
#7	MeSH descriptor: [Adrenergic beta-Antagonists] explode all trees	Advanced	5,661
#8	("beta-Adrenergic Blocker" OR "beta-Antagonists, Adrenergic" OR "Adrenergic beta Antagonists" OR "Blockader, Adrenergic beta-Receptor" OR "Antagonists, beta-Adrenergic" OR "beta-Adrenergic Antagonists" OR "beta-Adrenergic Blocking Agents" OR "beta-Receptor Blockaders, Adrenergic" OR "beta Adrenergic Blocker" OR "beta Adrenoceptor Antagonists" OR "beta Adrenoceptor Antagonist" OR "beta Blockers, Adrenergic" OR " Blockaders, Adrenergic beta-Receptor"):ti,ab,kw	Advanced	5,783
#9	#7 OR #8	Advanced	6,023
#10	#3 AND #6 AND #9	Advanced	208

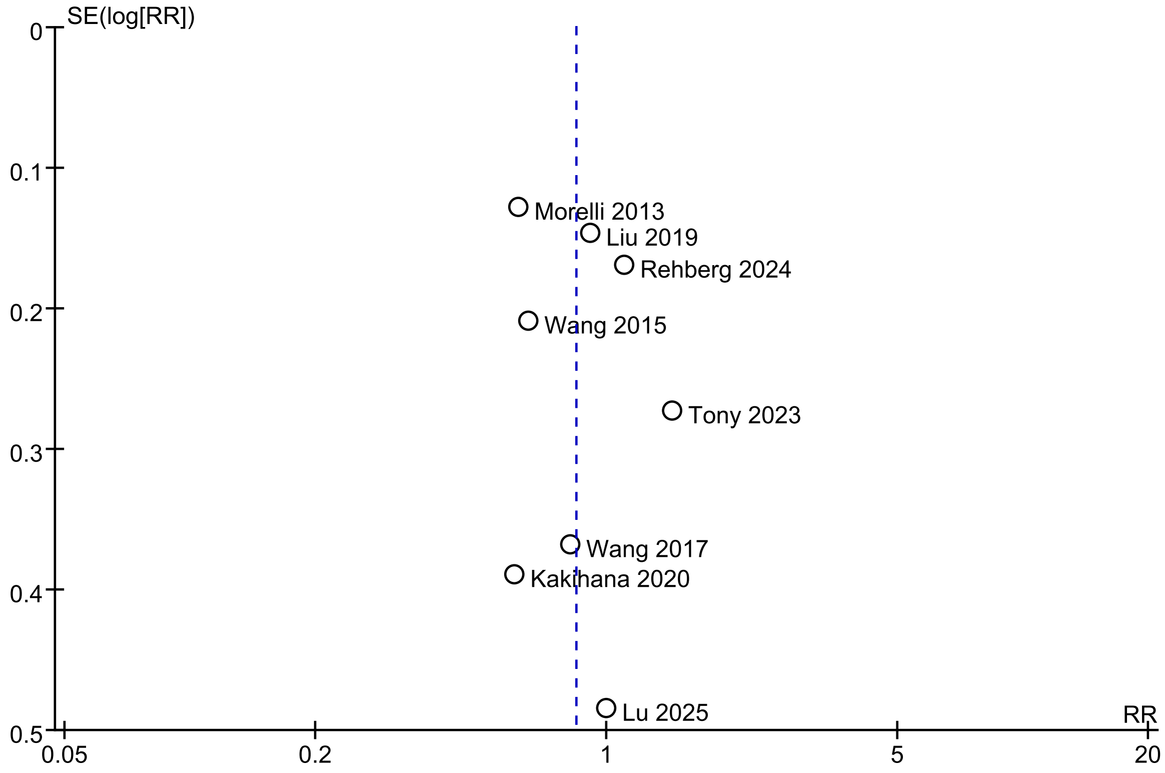

Figure S1. Funnel plot
